# Aerobic exercise increases brain vessel lumen size and blood flow in young adults with elevated blood pressure. Secondary analysis of the TEPHRA randomized clinical trial

**DOI:** 10.1016/j.nicl.2023.103337

**Published:** 2023-01-24

**Authors:** Winok Lapidaire, Nils D. Forkert, Wilby Williamson, Odaro Huckstep, Cheryl MJ Tan, Maryam Alsharqi, Afifah Mohamed, Jamie Kitt, Holger Burchert, Pauline Mouches, Helen Dawes, Charlie Foster, Thomas W. Okell, Adam J. Lewandowski, Paul Leeson

**Affiliations:** a Oxford Cardiovascular Clinical Research Facility, Division of Cardiovascular Medicine, Radcliffe Department of Medicine, University of Oxford, Oxford, United Kingdom; b Department of Radiology and Hotchkiss Brain Institute, University of Calgary, Calgary, Alberta, Canada; c School of Medicine, Trinity College Dublin, Dublin, Ireland; d Department of Cardiac Technology, College of Applied Medical Sciences, Imam Abdulrahman Bin Faisal University, Dammam, Saudi Arabia; e Department of Diagnostic Imaging and Radiotherapy, Faculty of Health Sciences, Universiti Kebangsaan Malaysia, Malaysia; f NIHR Exeter BRC, Medical School, University of Exeter, Exeter, United Kingdom; g Bristol Medical School, University of Bristol, Bristol, United Kingdom; h Wellcome Centre for Integrative Neuroimaging (FMRIB), Nuffield Department of Clinical Neurosciences, University of Oxford, Oxford, United Kingdom; i Life Sciences Research Center, Department of Biology, United States Air Force Academy, United States; j Department of Sport, Exercise and Health, University of Basel, Basel, Switzerland; k Ludwig Institute for Cancer Research, Nuffield Department of Medicine, University of Oxford, Oxford OX3 7DQ, UK

**Keywords:** Hypertension, Exercise intervention, Cerebral artery, Cerebral blood flow, Young adult, MRI, CBF, Cerebral Blood Flow, SBP, Systolic Blood Pressure, DBP, Diastolic Blood Pressure, TOF, Time-of-Flight, ASL, Arterial Spin Labelling, ICA, Internal Carotid Artery, MCA, Middle Cerebral Artery, PCA, Posterior Cerebral Artery, ACA, Anterior Cerebral Artery, BA, Basilar Artery

## Abstract

**Importance:**

Cerebrovascular changes are already evident in young adults with hypertension and exercise is recommended to reduce cardiovascular risk. To what extent exercise benefits the cerebrovasculature at an early stage of the disease remains unclear.

**Objective:**

To investigate whether structured aerobic exercise increases brain vessel lumen diameter or cerebral blood flow (CBF) and whether lumen diameter is associated with CBF.

**Design:**

Open, parallel, two-arm superiority randomized controlled (1:1) trial in the TEPHRA study on an intention-to-treat basis. The MRI sub-study was an optional part of the protocol. The outcome assessors remained blinded until the data lock.

**Setting:**

Single-centre trial in Oxford, UK.

**Participants:**

Participants were physically inactive (<150 min/week moderate to vigorous physical activity), 18 to 35 years old, 24-hour ambulatory blood pressure 115/75 mmHg-159/99 mmHg, body mass index below 35 kg/m^2^ and never been on prescribed hypertension medications. Out of 203 randomized participants, 135 participated in the MRI sub-study. Randomisation was stratified for sex, age (<24, 24–29, 30–35 years) and gestational age at birth (<32, 32–37, >37 weeks).

**Intervention:**

Study participants were randomised to a 16 week aerobic exercise intervention targeting 3×60 min sessions per week at 60 to 80 % peak heart rate.

**Main outcomes and Measures:**

cerebral blood flow (CBF) maps from ASL MRI scans, internal carotid artery (ICA), middle cerebral artery (MCA) M1 and M2 segments, anterior cerebral artery (ACA), basilar artery (BA), and posterior cerebral artery (PCA) diameters extracted from TOF MRI scans.

**Results:**

Of the 135 randomized participants (median age 28 years, 58 % women) who had high quality baseline MRI data available, 93 participants also had high quality follow-up data available. The exercise group showed an increase in ICA (0.1 cm, 95 % CI 0.01 to 0.18, p =.03) and MCA M1 (0.05 cm, 95 % CI 0.01 to 0.10, p =.03) vessel diameter compared to the control group. Differences in the MCA M2 (0.03 cm, 95 % CI 0.0 to 0.06, p =.08), ACA (0.04 cm, 95 % CI 0.0 to 0.08, p =.06), BA (0.02 cm, 95 % CI −0.04 to 0.09, p =.48), and PCA (0.03 cm, 95 % CI −0.01 to 0.06, p =.17) diameters or CBF were not statistically significant. The increase in ICA vessel diameter in the exercise group was associated with local increases in CBF.

**Conclusions and Relevance:**

Aerobic exercise induces positive cerebrovascular remodelling in young people with early hypertension, independent of blood pressure. The long-term benefit of these changes requires further study.

**Trial Registration:**

Clinicaltrials.gov NCT02723552, 30 March 2016

## Highlights

• Exercise intervention increased internal carotid and middle cerebral artery lumen.
• Exercise intervention did not change CBF.
• Increased ICA lumen diameter was associated with a regional increase in CBF.
• Cerebrovasculature is modifiable in young adults with high blood pressure.

## Graphical abstract

**Figure:**
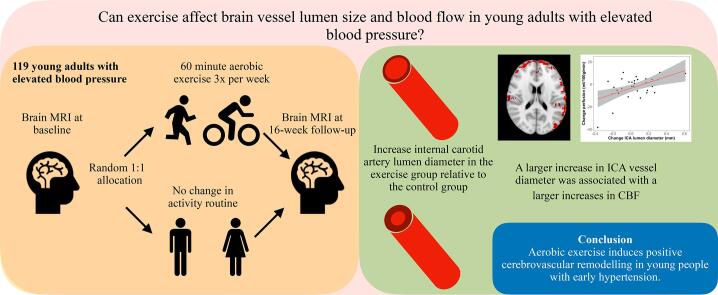

## 1 Introduction

Hypertension is the main risk factor for cardiovascular and cerebrovascular diseases (Ezzati et al., 2002, O’Donnell et al., 2010, Yusuf et al., 2004), responsible for half of strokes (O’Donnell et al., 2010), and is increasing in prevalence globally (World Health Organization, 2013). We have shown hypertensive cerebrovascular changes, pathologically-related to later life events, are already established in young adulthood (Bancks et al., 2017, Magnussen, 2017, Marcon et al., 2019, O’Leary et al., 1999, Williamson et al., 2018). Once present, the increased wall thickness and reduced lumen diameter can limit ability for vessel dilatation to maintain cerebral blood flow (CBF) (Brown et al., 2018, Claassen et al., 2021, Elsaid et al., 2021). As a result, vascular autoregulation leads to an increase in central blood pressure, exacerbating the hypertensive disease progression (Brown et al., 2018, Claassen et al., 2021, Elsaid et al., 2021). CBF becomes more difficult to maintain as hypertensive disease progresses (Muller et al., 2012), increasing susceptibility to hypoperfusion (Iadecola and Gottesman, 2019), risk of white matter damage (Aribisala et al., 2014, Brisset et al., 2013) and clinical sequelae, such as dementia (Czakó et al., 2020). Interventions during the first decades of life to prevent the early stages of cerebrovascular remodelling may therefore be required to slow disease development.

Current guidelines for hypertension recommend physical activity to reduce cardiovascular risk (Pescatello et al., 2015), but we have recently demonstrated exercise training has minimal impact on blood pressure in young adults during early stages of elevated blood pressure (Williamson et al., 2022) Click or tap here to enter text. Nevertheless, exercise can affect conduit artery vascular tone (Barnes et al., 2018), endothelial function (Pedralli et al., 2018), and vessel size (Dinenno et al., 2001, Naylor et al., 2006). Furthermore, regular exercise over longer periods in older adults can slow ageing-related reduction in CBF and blood velocity (Nishijima et al., 2016) to delay brain ageing and degenerative diseases (di Liegro et al., 2019). To investigate whether physical exercise could counteract early effects of hypertension on brain vessel lumen size and CBF, independent of blood pressure, we imaged the cerebrovasculature in young adults with elevated blood pressure participating in a randomized controlled trial that compared the effect of a 16-week exercise intervention with usual care in young adults with elevated blood pressure.

## 2 Methods

### 2.1 Study participants

The TEPHRA trial protocol is available in Supplement 1. The trial design and the results of the primary outcomes have been described previously (Williamson et al., 2022). This was a single-centre, open, two-arm, parallel superiority randomized (1:1) controlled trial. All participants were invited to complete the optional MRI protocol until 100 participants joined the MRI substudy. Thereafter, only preterm-born participants were invited to complete the MRI protocol to ensure sufficient heterogeneity in birth history. After completion of the baseline study visit, participants were randomly assigned to an aerobic training and physical activity intervention group or a control group in a 1:1 ratio, stratified for sex, age (<24, 24–29, 30–35 years) and gestational age at birth (<32, 32–37, >37 weeks) using Sealed EnvelopeTM. Researchers responsible for data analysis were blinded until data acquisition and cleaning was done. Participants in the intervention arm were asked to complete three aerobic training sessions per week, aiming for 60 min exercise at 60–80 % peak heart rate measured at baseline. The number of sessions attended and activity measurements from a wrist-worn activity monitor were recorded. Participants in the control group were given educational materials on hypertension, hypertension prevention and recommended lifestyle behaviours for cardiac health. Eligibility criteria included: age 18 to 35 years; 24-hour awake ambulatory systolic (SBP) and/or diastolic (DBP) blood pressure > 115/75 mmHg and < 159/99 mmHg; body mass index < 35 kg/m^2^; no history of hypertension medication prescription; verifiable birth history of preterm birth; internet access. Exclusion criteria were: pregnancy; participation in structured exercise more than once per week or high cardiovascular fitness; not able to provide consent; contra-indications to exercise; not able to walk briskly on the flat for 15 min; evidence of significant cardiovascular disease. Brain MR scans were acquired on a 3.0 T scanner (Siemens, Munich, Germany) at baseline and at 16 weeks. Participants gave written informed consent. The study was approved by the University of Oxford as host institution and study Sponsor and the South Central Research Ethics Committee for the National Health Service Health Research Authority (Reference 16/SC/0016). Enrolment occurred between June 30, 2016, and October 26, 2018 and final follow-up of the trial ran until January 9, 2020. Trial data and materials are available to be shared subject to data sharing agreement and will be reviewed on a case-by-case basis. Please contact the corresponding author for requests.

### 2.2 MRI study and acquisition

The scan protocol included a T1-weighted structural scan (TR/TE = 2040/4.7 ms, flip angle 8°, FOV 200 mm, voxel size 1.0 mm isotropic), Time-of-Flight (TOF) MRA (TR/TE = 23/8 ms, flip angle 10°, FOV 300 mm voxel size 1.6×1.2×5.0 mm) and multi-delay vessel-encoded pseudocontinuous Arterial Spin Labelling (ASL; TR/TE = 4.05/14 ms, voxel size 3.4x3.4x5 mm, post-labeling delays 0.25, 0.5, 0.75, 1.0, 1.25, 1.5 s)(Okell et al., 2013).

### 2.3 MRI processing

T1-weighted images were processed using the FSLanat pipeline (Smith et al., 2004)^33^ to create grey matter segmented images and a T1 to the MNI registration matrix. Vessel segmentation in TOF MRA datasets was performed using an automated segmentation algorithm (Forkert et al., 2012, Forkert et al., 2011), from which vessel lumen diameter in mm were determined for pre-determined arteries (ICA, MCA M1 and M2, ACA, BA, PCA), averaged between the left and right side (Mouches and Forkert, 2019). All vessel segmentation results were visually checked and manually corrected if required. Participants were removed from the analysis if outlier data (three standard deviations above or below the mean) was observed in the baseline visit or the 16-week visit. ASL image quality was improved by independent component analysis noise reduction (Carone et al., 2019) prior to processing using the OXASL pipeline for vessel-encoded images to obtain parametric maps of tissue CBF in ml/100 g/min (Chappell et al., 2012, Chappell et al., 2009) and CBV in mL blood/100 ml in the grey matter for the whole brain as well as for each of the four encoded vessels (Okell et al., 2013), reflecting the left and right vertebral and internal carotid arteries in the neck. The CBF maps were calibrated for cross-subject comparison using a single M0 value, derived from the cerebrospinal fluid (CSF) in the ventricles. A mask map was created that indicated which voxels were predominantly (>50 % of CBF) supplied by the left and right vertebral arteries and a mask map was created that indicated which grey matter voxels were predominantly supplied by the left and right internal carotid arteries.

All ASL images were registered to their corresponding T1 scan and then registered to MNI space using T1 to the MNI registration matrix. For each participant, the 16-week ASL maps in MNI space were then subtracted from the corresponding baseline ASL map in MNI space to calculate the change in ASL parameters in each voxel during the exercise intervention period.

### 2.4 Outcomes

The primary outcomes were the change in vessel lumen diameter of the ICA, MCA M1 and M2, ACA, BA, PCA and voxel-wise brain CBF from baseline to follow-up. The relationship between the significant effects of exercise in vessel lumen diameter parameters and changes in CBF in the exercise intervention group was an exploratory outcome.

### 2.5 Statistical analysis

Statistical analyses were run in R (version 4.0.3). Group differences in vessel lumen diameter at follow-up between the exercise intervention group and control group were examined using analysis of covariance (ANCOVA) adjusting for baseline values of vessel lumen diameter and sex, age, and gestational age (<32, 32–37, >37 weeks). FSL’s randomise function was used to run voxel-based analyses brain maps to detect group differences in changes in CBF between baseline and follow-up and the association between vessel lumen diameter changes. Analyses were run with the vertebral artery supply mask (PCA), or the internal carotid artery supply mask (ICA, MCA M1, MCA M2, ACA). CBF values were averaged across voxels with significant associations between vessel lumen diameter and CBF changes over the exercise intervention period, and associations between vessel diameter changes and mean CBF values were investigated with a Pearson’s correlation analysis.

## 3 Results

### 3.1 Study population

**Fig. 1:**
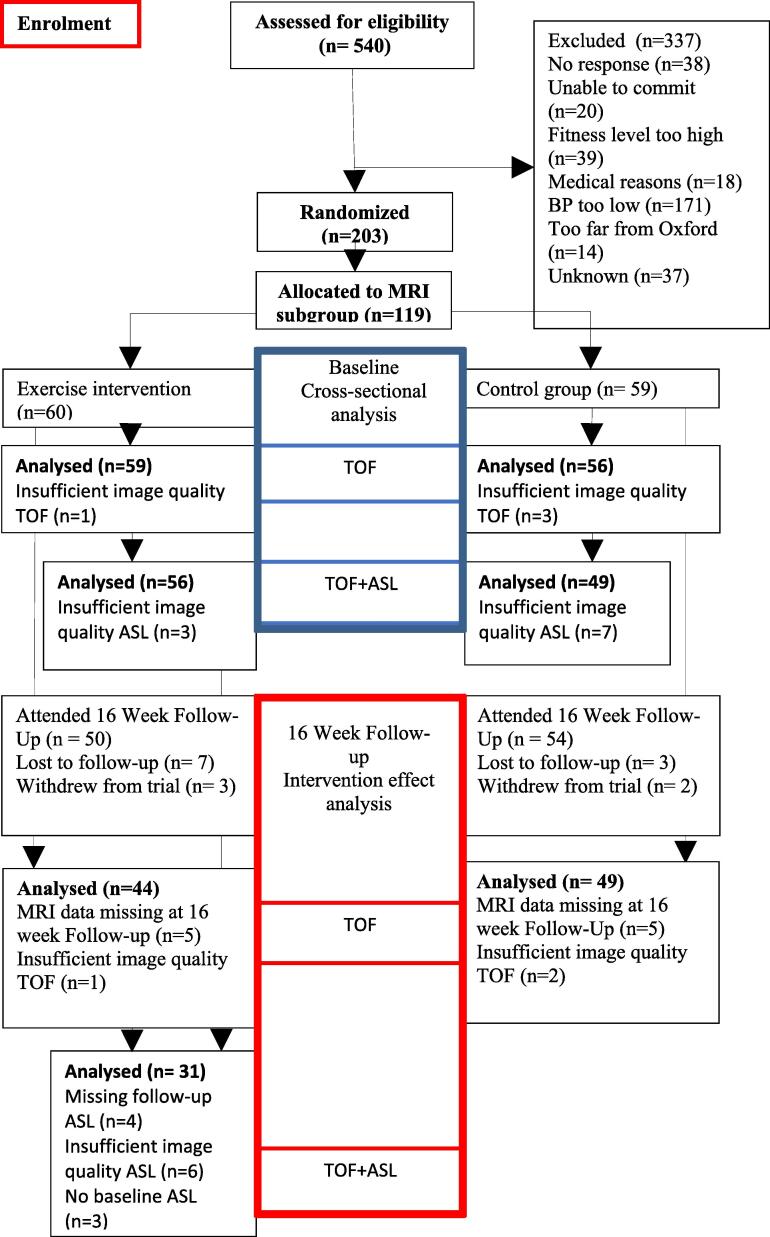
Overview of the study, inclusion and exclusion criteria for the intervention and MRI processing.

Of the 119 randomized participants who took part in the MRI sub-study, 100 (84 %) returned for 16-week follow-up. Fig. 1 shows the flow through the study and the number of TOF and ASL scans available for analysis.

There were no significant differences in baseline characteristics between the randomised groups of participants included in the MRI substudy vessel lumen diameter analysis (Table 1) or between participants included and excluded in the final analysis at baseline (Table 2). In the sample analysed here, there was a statistically significant increase in Peak VO2 over the intervention period in the exercise intervention group (0.23 ml/kg/min, 95 % CI 0.12 to 0.33, p < 0.001). There were two adverse events reported in the training group; an ankle sprain and a toe injury.

**Table 1:** Characteristics at baseline of participants included in the MRI substudy vessel lumen diameter analysis.

	Exercise Intervention n = 44	Control n = 49
Age, Mean (SD)	28.2 (4.4)	27.8 (4.4)
Male, No. (%)	23 (52)	25 (51)
Preterm, No. (%)	19 (43)	18 (37)
Employed, No. (%)	41 (98)	45 (96)
University Degree, No. (%)	32 (76)	40 (85)
Family history of high blood pressure, stroke, or heart attack, No. (%)	19 (43)	17 (35)
BMI, Mean (SD)	24.5 (3.1)	25.1 (3.4)
Smoker, No. (%)	0 (0)	3 (6)
Units of alcohol per week, Mean (SD)	6.0 (5.6)	5.1 (7.7)
24 h awake ambulatory systolic blood pressure, Mean (SD)	129.7 (11.0)	128.2 (8.5)
24 h awake ambulatory diastolic blood pressure, Mean (SD)	77.4 (6.8)	77.5 (7.1)
Oxygen uptake peak, Mean (SD)	34.4 (7.1)	35.1 (7.4)
Cholesterol HDL ratio, Mean (SD)	3.5 (0.9)	3.4 (1.2)
Glucose mmoll, Mean (SD)	4.8 (0.4)	4.9 (0.4)
HOMA insulin resistance, Mean (SD)	0.9 (0.4)	0.9 (0.4)

**Table 2:** Characteristics at baseline of participants included and excluded in the TOF, and TOF-ASL analyses.

	Participants included in TOF analysis n = 93	Participants not included in TOF analysis n = 110	Participants included in TOF + ASL analysis – Exercise group only n = 31	Participants not included in TOF + ASL analysis n = 172
Age, Mean (SD)	28.0 (4.4)	27.5 (3.9)	28.2 (4.3)	27.7 (4.1)
Male, No. (%)	48 (52)	49 (45)	13 (42)	84 (49)
Preterm, No. (%)	56 (60)	95 (86)	17 (55)	134 (78)
Employed, No. (%)	3 (3)	0 (0)	1 (3)	2 (1)
University Degree, No. (%)	72 (81)	83 (78)	22 (76)	133 (80)
Family history of high blood pressure, stroke, or heart attack, No. (%)	0.4 (0.5)	0.5 (0.5)	0.4 (0.5)	0.4 (0.5)
BMI, Mean (SD)	24.8 (3.3)	25.2 (3.8)	24.2 (3.5)	25.2 (3.6)
Smoker, No. (%)	3 (3)	10 (9)	0 (0)	13 (8)
Units of alcohol per week, Mean (SD)	5.5 (6.8)	6.0 (7.5)	5.2 (5.2)	5.9 (7.5)
24 h awake ambulatory systolic blood pressure, Mean (SD)	128.9 (9.7)	128.5 (8.4)	127.5 (9.3)	128.9 (8.9)
24 h awake ambulatory diastolic blood pressure, Mean (SD)	77.5 (6.9)	77.0 (6.9)	77.2 (6.7)	77.2 (7.0)
Oxygen uptake peak, Mean (SD)	34.8 (7.2)	33.1 (7.4)	33.9 (7.2)	33.8 (7.4)
Cholesterol HDL ratio, Mean (SD)	3.4 (1.1)	3.3 (1.3)	3.5 (0.9)	3.3 (1.3)
Glucose mmoll, Mean (SD)	4.8 (0.4)	4.8 (0.7)	4.8 (0.4)	4.8 (0.6)
HOMA insulin resistance, Mean (SD)	0.9 (0.4)	1.2 (1.0)	0.9 (0.4)	1.1 (0.9)

### 3.2 Exercise intervention effect on vessel lumen size and CBF

Relative to the control group, the increase in ICA and MCA M1 vessel lumen diameter was significantly greater between baseline and 16-week measurements in the exercise group than in the control group (ICA mean between-group difference: 0.1 mm [95 % CI, 0.01–0.18]; MCA M1 mean group difference: 0.05 mm [95 % CI, 0,01–0.10]). There were no significant group differences in the change in MCA M2, ACA, BA, and PCA (Table 3).

**Table 3:** Vessel lumen diameter change with exercise per vessel segment.

	Exercise Intervention		Control			
	*n*	*Mean (SD)*	*n*	*Mean (SD)*	*Mean difference (95 % CI) adjusted*	*p-value*
ICA						
*Baseline*	44	1.82 (0.27)	49	1.86 (0.28)	*–*	*–*
*16 weeks*	44	1.85 (0.29)	49	1.78 (0.30)	0.1 (0.01,0.18)	0.03

MCA_M1						
*Baseline*	44	1.03 (0.14)	49	1.03 (0.15)	*–*	*–*
*16 weeks*	44	1.05 (0.13)	49	0.99 (0.17)	0.05 (0,01,0.10)	0.03

MCA_M2						
*Baseline*	44	0.73 (0.08)	49	0.74 (0.09)		
*16 weeks*	44	0.74 (0.09)	49	0.72 (0.11)	0.03 (0,0.06)	0.08

ACA						
*Baseline*	44	0.78 (0.12)	49	0.80 (0.11)		
*16 weeks*	44	0.79 (0.11)	49	0.76 (0.13)	0.04 (0.0,0.08)	0.06

BA						
*Baseline*	44	1.09 (0.23)	49	1.08 (0.18)		
*16 weeks*	44	1.1 (0.19)	49	1.07 (0.23)	0.02 (-0.04,0.09)	0.48

*PCA*						
*Baseline*	44	0.83 (0.11)	49	0.82 (0.11)		
*16 weeks*	44	0.82 (0.10)	49	0.78 (0.13)	0.03 (-0.01,0.06)	0.17

There were no voxel-wise differences between randomised groups in the change in CBF during the exercise intervention period.

### 3.3 Relationship between vessel lumen diameter and CBF exercise intervention changes

**Fig. 2:**
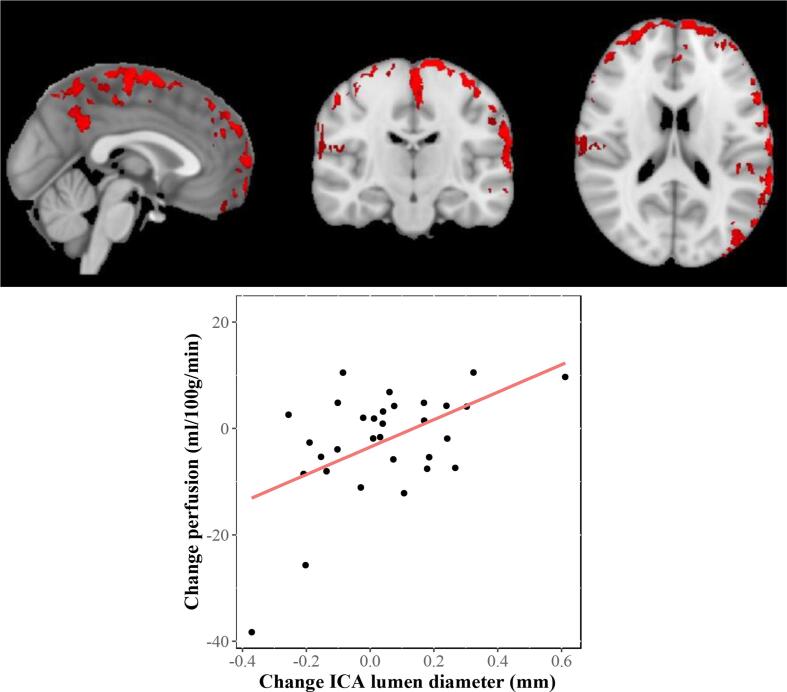
Top: voxel-wise positive association between change in vessel lumen diameter and ASL parameters with exercise, significant voxels indicated in red. Bottom: perfusion change averaged over the significant voxels plotted against the change in ICA lumen diameter (bottom). (For interpretation of the references to colour in this figure legend, the reader is referred to the web version of this article.)

This exploratory analysis showed that an increase in ICA lumen diameter between study visits was associated with an increase CBF in the exercise intervention group in the left and right lateral parietal lobe (Fig. 2). In the significant clusters, a millimetre increase in ICA lumen diameter was associated with a mean 0.5 ml/100 g/min [t(29) = 3.3, 95 % CI, 0.20–0.74, p = 0.003].

There were no significant associations between MCA M1 lumen diameter and CBF changes over the exercise intervention period. The association between the change in ICA lumen diameter and perfusion is still significant when examined with a non-parameteric Spearman test (S(29) = 3074, ρ = 0.04, p = 0.04).

## 4 Discussion

### 4.1 Exercise intervention effects

This randomized controlled 16-week intervention trial supports the hypothesis that the cerebrovasculature is sensitive to exercise adaptation early in hypertension, independent of blood pressure changes, and exercise may help counteract hypertension-induced adverse remodelling. In this study, young adults with elevated blood pressure showed on average an increase in ICA and MCA M1 lumen diameter following supervised aerobic exercise. The increase in ICA lumen diameter over the course of the exercise intervention was associated with local increases in CBF.

Risk of vascular events and death increases at both extremes of brain arterial diameter sizes (Gutierrez et al., 2015). The young adults with elevated blood pressure in this study had a below average vessel lumen diameter for their age (Mouches and Forkert, 2019). Although inward remodelling and increased vagal tone in response to hypertension protects the brain against the damaging effects of high blood pressure to the downstream microcirculation, it also increases the susceptibility to ischemic injury (Brown et al., 2018). The exercise intervention in this study, increased vessel lumen diameter by 5.5 % (ICA) and 4.9 % (MCA M1) when adjusting for covariates and thus counteracted the reductions in vessel diameter observed in this group (Brown et al., 2018, Green et al., 2012, Mulvany et al., 1978). If sustained, it may therefore have a protective effect against white matter lesions and grey matter atrophy that is observed after vascular inward remodelling. Interestingly, the effects of exercise training on vessel lumen diameter were strongest in the ICA and MCA M1, large cerebral arteries most proximal to the heart (Mouches and Forkert, 2019, Zarrinkoob et al., 2015). These vessels are exposed to the largest increase in shear stress from blood flow changes during exercise (Hellsten and Nyberg, 2016) and this dynamic shear stress and transmural pressure (Aalkjaer et al., 1987, Brown, 2003, Hellsten and Nyberg, 2016, Lundgren et al., 1974) could account for the exercise response (Hellström et al., 1996, Moraine et al., 1993, Nishijima et al., 2016, Querido and Sheel, 2007).

There were no significant differences in voxel-wise CBF at rest between the exercise intervention group and the control group. Previous studies investigating relations between physical activity and CBF show mixed results. A *meta*-analysis on studies measuring CBF with MRI on populations across a large age range showed that exercise training had little effect on global CBF and a varying effect on regional CBF (Smith et al., 2021). However, in our study, an increase in ICA vessel lumen diameter over the course of the exercise intervention was associated with a graded increase in CBF. This effect was small with a 0.1 mm increase in ICA lumen diameter associated with just a 0.05 ml/100 g/min increase in CBF in the significant clusters. This may explain the lack of difference at a group level as our sample size was underpowered to identify this degree of mean difference in CBF between intervention and usual care. The increase in CBF in regions around the edge of the brain could potentially be due to a change in pial vessel diameter. Cerebral pial (resistance) vessels are known to respond quickly to a low pH induced by exercise, and exercise-induced hyperventilation (Querido and Sheel, 2007). There is little research on the effect of pial vessels on exercise in humans, but in mice, exercise prevented age-induced rarefaction of pial collaterals (Rzechorzek et al., 2017). Exercise has also been found to improve impaired eNOS- and nNOS-dependent dilation of pial arterioles in rats (Arrick et al., 2014, Mayhan et al., 2010).

In the whole trial, no significant changes in blood pressure were seen in response to exercise (Williamson et al., 2022) despite a significant increase in cardiovascular fitness. Epidemiological evidence indicates that >40 % of the beneficial effect of exercise on cardiovascular disease cannot be explained by modification of risk factors (Mora et al., 2007) and additional vascular adaptations to exercise independent of any change in blood pressure could explain part of the benefit. Reduced lumen diameter is associated with increased risk of stroke and dementia in older cohorts. Therefore, if the relative increase in vessel diameter observed in this study was maintained to later life, the impact could be significant on the prevention of stroke and dementia. The delay of dementia onset by a few years can have substantial impact on morbidity and mortality (Mora et al., 2007, Winblad et al., 2016, Wolters et al., 2019).

### 4.2 Limitations and future research

Despite our comprehensive inclusion and exclusion criteria, our cohort of young adults with elevated blood pressure might have had considerable heterogeneity in the cause and duration of their high blood pressure. Some of the variation in vessel size could relate to heterogeneity in risk factors between participants induced by the exercise intervention and a larger study will be able to explore in subgroups with different risk factors. Specifically, adults born preterm have a unique cardiac phenotype (Lewandowski et al., 2013), which may affect their response to exercise. Since recruitment strategy aimed at including individuals born preterm, the proportion of people born preterm is higher in this study than in the general population.

Since the CBF images were registered to a brain template for voxel-wise analyses, these analyses could be affected by registration errors. Nevertheless, the results could only have been caused by a registration error if the amount of increase in ICA lumen diameter in response to an exercise intervention was associated with the registration to the average template. The study was also performed in young people and therefore long term follow up with clinical event rates is not feasible. A longitudinal follow-up study could investigate if changes in vascular lumen diameter and blood flow and volume in the exercise intervention group remained over time. Research remains to be done to establish the optimal exercise type, dose, and duration to protect brain health in young adults with elevated blood pressure and whether resting CBF can be changed through exercise. A follow-up study could also examine the effect of exercise on white matter lesions. The intervention period was too short to be able to observe group differences in lesion changes in this study.

## 5 Conclusion

Exercise may play a role in protecting long-term brain health in young people with elevated blood pressure, even when there is no reduction in blood pressure. Future studies should investigate whether sustained exercise could decrease hypertension-related stroke and cognitive decline later in life.

## Declaration of Competing Interest

The authors declare that they have no known competing financial interests or personal relationships that could have appeared to influence the work reported in this paper.

## Data Availability

Data will be made available on request.
